# GEOTrat Points as a free resource in QGIS for mapping the performance of agricultural experiments

**DOI:** 10.1371/journal.pone.0332751

**Published:** 2025-10-03

**Authors:** Laura Cristina Moura Xavier, George Deroco Martins, Guilherme Pereira de Oliveira, Murillo Guimarães Carneiro

**Affiliations:** 1 Graduate Program in Geography, Federal University of Uberlândia, Uberlândia, Minas Gerais, Brazil; 2 Graduate Program in Agriculture in Geospatial Information, Federal University of Uberlândia, Monte Carmelo, Minas Gerais, Brazil; 3 Instituto Federal Goiâno, Ciêncais Agrárias, Rio Verde, Goiás, Brazil; Central Marine Fisheries Research Institute, INDIA

## Abstract

Agricultural experimentation requires careful selection of the experimental design and model for analyzing treatment data. However, even with rigorous experimental control, the discrepancies between treatments are so subtle that traditional statistical models fail to highlight statistically significant differences that occur in field practice. The incorporation of geotechnologies offers the ability to map agricultural variability, but a gap still exists in the availability of tools designed to map and evaluate the effectiveness of agricultural experiments. To overcome this limitation and promote the wider application of Geographic Information Systems in agriculture, the scope of this study focuses on the development of a resource in QGIS software, aimed at evaluating agricultural experiments using a randomized block design with up to five treatments. The resource developed incorporates spatial interpolation techniques using geostatistical kriging, map generation, and statistics. The study used yield samples from six different crops to identify quantitative and spatial differences between two-treatment experiments in terms of yield gain. The performance evaluation included statistical measures such as Root Mean Square Error (RMSE) and Pearson’s correlation coefficient to validate the accuracy of interpolated surfaces. The results consisted of two surfaces representing the study area treated with each of the treatments, as well as a surface reflecting the yield gain of the reference treatment in relation to the control treatment, accompanied by relevant descriptive statistics measures on this gain surface. The experiments showed that RMSE varied between 4.6% and 69.71%, depending on the crop and treatment, while Pearson’s correlation ranged from −0.16 to 0.86, indicating varying degrees of agreement between interpolated and observed data. The tool successfully generated yield gain maps, allowing spatial visualization of treatment differences, with an accuracy of up to 95.40% in detecting spatial and numerical variations between treatments. The tool, called GEOTrat - Points, offers the flexibility to evaluate agricultural experiments of various designs, encompassing different crops and different quantities of samples, providing both numerical and spatial analysis.

## Introduction

Agricultural experimentation is fundamental to the development and improvement of agricultural production. It involves the application of scientific methods to test and evaluate different techniques, practices, and materials used in the production of food and agricultural products. Agricultural experiments can be conducted at various scales, ranging from laboratory studies to large-scale field experiments. In addition, they can include testing different crop varieties, fertilizers, irrigation techniques, cultivation systems, and other factors that affect agricultural production [1].

One of the most important aspects in conducting agricultural research experiments is the appropriate choice of the experimental design and data analysis model. This ensures randomization, replication, and control of treatments [2], guaranteeing independence between experimental units and increasing the accuracy of estimates [3]. However, even with this experimental control, the differences between treatments are so subtle that classical statistical models cannot show significant differences in the study variable(s) [4–5]. This is because these models do not account for spatial variations in environmental and soil factors or the spatial dependence within plots.

Although the use of geotechnologies enables the mapping of agricultural variability, their application in field experimentation still faces significant challenges. The main difficulty lies in detecting subtle differences between treatments, as environmental factors such as soil variability and microclimate conditions can either mask or amplify the effects of tested inputs. Additionally, traditional statistical analysis methods often do not account for spatial dependence among experimental units, which can compromise accuracy in identifying treatment response patterns. Existing market solutions, such as precision agriculture software, generally support agricultural monitoring but are not specifically designed for the detailed analysis of field experiments with multiple treatments. As a result, there is a growing need for tools that integrate advanced geoprocessing techniques and spatial statistics to assess treatment effectiveness more robustly. The tool proposed in this study aims to fill this gap by enabling the visualization and spatial quantification of differences between treatments, promoting a more precise approach to agricultural experimentation and decision-making.

The application of geographic positioning technologies in agricultural experimentation enables the collection of georeferenced information, allowing farmers to conduct experiments more accurately. Geotechnologies encompass the use of technologies such as satellite and aircraft remote sensing, Geographic Information Systems (GIS) and global positioning systems [6]. Using this geospatial information, it is possible to map the spatial variability of factors such as soil, water, and nutrients and the incidence of pests and diseases. It is also possible to monitor crop growth, helping to reduce costs, optimize the use of resources, and minimize environmental impacts [7].

Technologies such as GIS play an important role in modern agriculture, allowing farmers to collect, manage, and analyze geospatial data related to agricultural production [8]. QGIS, free software with open-source code, is a GIS platform that enables the development of tools to extend its functionalities and that allows the search and installation of resources and plugins developed by third parties [9]. However, the lack of comprehensive tools for mapping and evaluating the effectiveness of agricultural experiments—especially those that integrate spatial analysis, geoprocessing, and visualization—is still a limitation [10].

In the current landscape of precision agriculture software, solutions with various applications are available, such as FarmWorks by Trimble, AgLeader SMS, FarmLogs, Agrian, and Climate FieldView, which, although not specifically for this purpose, can be used to analyze agricultural experiments. Within the QGIS environment itself, although some plugins are dedicated to precision agriculture, such as Precision Agriculture Tools – PAT, GeoDataFarm, Smart Map, and others (see QGIS Python Plugins Repository: plugins tagged with agriculture https://plugins.qgis.org/plugins/tags/agriculture/?per_page=100), these options often lack a complete integration of advanced techniques for evaluating agricultural experiments.

To overcome this limitation and promote the wider application of GIS in agriculture, the aim of this work was to develop a resource for QGIS software for evaluating agricultural experiments with a randomized block design of up to five treatments. This resource uses spatial interpolation techniques to model agricultural variables of interest to the user, statistical analysis, and the generation of maps, allowing a quantitative and spatial comparison of the treatments used. In this research, we used yield data to investigate whether the integration of these technologies and methods can offer an effective solution to complement the evaluation of agricultural experiments, facilitating the identification of substantial differences between treatments in yield gain.

## Resource development

### Proposal, availability and interface

The tool developed in this study was called GEOTrat - Points, and its most up-to-date version is available in the GitHub repository, accessible online via the link https://github.com/LauraMouraXavier/geotrat (accessed in February 2024). The repository contains the GEOTrat_Points files with the model and.py extensions, compatible with QGIS Desktop version 3.22.8. To use the developed resource, it is necessary to import one of these files into the QGIS software’s processing toolbox and to install the SAGA and SAGA Next Gen add-ons. To install GEOTrat users must download the repository as a ZIP file and extract it to a folder on their computer. Then by clicking on the Python icon in the processing toolbox, users can add the scripts (Python code files with.py extention) to toolbox. For more details, see the REDME file with planned installation instructions, step-by-step usage guidelines, and requirements and dependencies. The plugin repository has a sample dataset for users to test and validate the tool.

**Fig 1:**
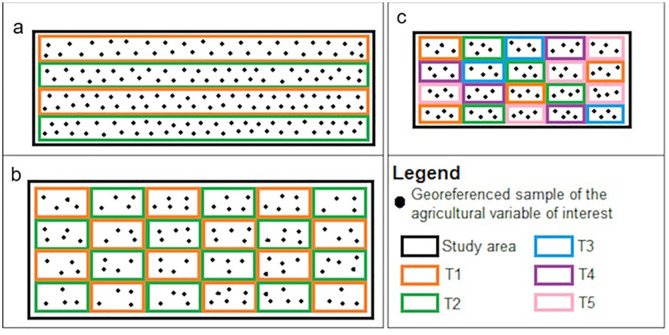
Examples of field experiment designs suitable for applying the tool developed, (a) example of a two-treatment continuous block design, (b) example of a two-treatment interleaved block design, and (c) example of a five-treatment interleaved block design.

The GEOTrat - Points tool is designed to evaluate the effectiveness of treatments quantitatively and spatially by generating maps through geostatistical interpolation. The agricultural experiment to be conducted in the field must be structured in a randomized block design, with a minimum of two treatments and up to five different treatments called T1, T2, T3, T4, and T5. The user, must collect georeferenced samples of the agricultural variable of interest to perform a comparative analysis. Fig 1 illustrates examples of field experiment design suitable for applying the resource developed in this study.

In addition to the examples of designs shown in Fig 1, both the study area and the treatment blocks are not restricted to specific dimensions and can take on any configuration. In addition, the number of samples is determined by the user, and it is suggested to follow the norms defined by agricultural experimentation manuals. More detailed information on the principles of experimental planning, design, and data analysis in agricultural contexts can be found in [11] and [12]. It is also suggested that the samples cover the entire study area, distributed homogeneously in equal quantities between the two treatments.

The fields displayed on the tool’s interface are for entering inputs and specifying the paths for storing the results. The inputs consist of information provided by the user, covering data related to the experiment conducted in the field as well as the parameters needed to conduct geostatistical interpolation. In the right-hand corner of the tool’s interface is a help text box with detailed information about each of the tool’s input and output parameters as well as details about the developers and versions available. The tool also has buttons for starting execution, closing the interface, accessing the help function, and running a set of batch processes.

The GEOTrat - Points tool consists of inputs, algorithms, and outputs. The following topics detail the specifications of the inputs, algorithms, and outputs generated by the tool.

### Inputs

The tool’s inputs must be supplied by the user and consist of vector layers, vector layer fields, coordinate reference system, numerical values, and enumerated lists of options. The specifications of the inputs and their formats are shown in Table 1.

**Table 1:** Input specifications and their formats.

Input Name	Format
Variable measured in the field	Vector Layer – Geometry type Point
Variable field	Vector Field – Data type Number
Treatment field	Vector Field – Data type String
Reference treatment	Enumerated List (T1 or T2)
Study area polygon	Vector Layer – Geometry type Polygon
Projected Coordinate System	Coordinate Reference System
Pixel size (m)	Number
Semivariogram model	Enumerated List (Linear, Exponential, Gaussian and Spherical)
Number of treatments	Number

To use the tool, the first piece of information to be entered by the user is the *variable measured in the field*, which must be a vector layer of point geometry in shapefile format. This vector layer should contain the georeferenced points of the samples obtained in the field experiment. The file’s attribute table must contain two mandatory fields: a numeric field with the values of the agricultural variable measured in the field, to be defined in the *Variable field* entry; and a textual field identifying the treatment applied (e.g., T1, T2, T3, T4, T5), to be defined in the *Treatment field* entry.

In the *Reference treatment* entry, which is an enumerated list with the options T1 (default), T2, T3, T4 and T5, the user must indicate which treatment will be considered the reference for comparison with the other treatments. In addition, in the *Number of treatments entry*, the user must indicate the number of treatments used; in this option, the minimum value is equal to 2 (default), and the maximum value is equal to 5.

In addition to the vector layer of points, the user must have a vector layer of polygon geometry in shapefile format, which will represent the delimitation of the field experiment’s study area. This vector layer must be inserted in the *Study area polygon* input.

The comparison of treatments conducted by the tool uses the technique of spatial interpolation using geostatistics, specifically the Kriging method (more details in section Algorithms). This method is used to generate maps by estimating matrix surfaces. Therefore, the user must provide additional information, such as the spatial resolution in meters of the generated surfaces, which must be specified in the *Pixel size (m)* input. It is recommended that the pixel size chosen is compatible with the average distance at which samples are collected in the field. In addition, in the *Semivariogram model* entry, the user must enter the mathematical model of the semivariogram to be used in kriging interpolation. The list provides the following options: Linear (default), Gaussian, Exponential, and Spherical. Once the inputs have been defined, the user can run the tool.

### Algorithms

The algorithms used to develop the tool belong to the QGIS geoprocessing package and the algorithm providers SAGA and GDAL. Table 2 shows the sequence of procedures conducted by the tool, detailing the order of execution, the description given to each process in the development of the tool, the algorithm used, and its respective provider.

**Table 2:** Procedures executed by the tool, order of execution, description, algorithm, and provider.

Order of execution	Process	Description in the tool	Algorithm	Provider
1	Reprojecting sample points	Reproject points	Reproject layer	QGIS
	Redesign area	Reproject area		
2	Rename the field of the agricultural variable of interest in the points layer	Rename variable field	Rename field	QGIS
	Rename the field specifying the point layer treatments	Rename treatment field		
3	Separate the points belonging to the different treatments	Separate T1	Extract by attribute	QGIS
		Separate T2		
		Separate T3		
		Separate T4		
		Separate T5		
4	Select 80% of the points belonging to each treatment	Select T1-80%	Rondom selection	QGIS
		Select T2-80%		
		Select T3-80%		
		Select T4-80%		
		Select T5-80%		
5	Extract the selected points to the vector layer	Extract T1-80%	Extract selected features	QGIS
		Extract T2-80%		
		Extract T3-80%		
		Extract T4-80%		
		Extract T5-80%		
6	Extract the remaining 20% of points to the vector layer	Extract T1-20%	Extract by location	QGIS
		Extract T2-20%		
		Extract T3-20%		
		Extract T4-20%		
		Extract T5-20%		
7	Estimate the variable of interest for the study area from 80% of the points -Interpolation by ordinary kriging	KrigO - T1-80%	Ordinary kriging	SAGA
		KrigO - T2-80%		
		KrigO - T3-80%		
		KrigO - T4-80%		
		KrigO - T5-80%		
8	Collect samples of the surface estimated with points using the 20% of the points	Sample T1	Sample raster values	QGIS
		Sample T2		
		Sample T3		
		Sample T4		
		Sample T5		
9	Calculate the estimation error of the generated surface	Error T1	Fied calculator	QGIS
		Error T2		
		Error T3		
		Error T4		
		Error T5		
10	Generate a surface of the calculated error – Interpolation by Ordinary Kriging	SupError T1	Ordinary kriging	SAGA
		SupError T2		
		SupError T3		
		SupError T4		
		SupError T5		
11	Add up the estimated and calculated error surfaces, generating a final estimated surface	T1	Raster calculator	GDAL
		T2		
		T3		
		T4		
		T5		
12	Cut out the final surface in the study area	T1_rec	Clip raster by mask layer	GDAL
		T2_rec		
		T3_rec		
		T4_rec		
		T5_rec		
13	Subtraction between final surfaces (Reference Treatment – Other Treatments)	Gain (T1 and T2)	Raster calculator	GDAL
		Gain (T1 and T3)		
		Gain (T1 and T4)		
		Gain (T1 and T5)		
		Gain (T2 and T3)		
		Gain (T2 and T4)		
		Gain (T2 and T5)		
		Gain (T3 and T4)		
		Gain (T3 and T5)		
		Gain (T4 and T5)		
14	Calculate basic surface statistics generated by subtraction	Stats (T1 and T2)	Raster layer statistics	QGIS
		Stats (T1 and T3)		
		Stats (T1 and T4)		
		Stats (T1 and T5)		
		Stats (T2 and T3)		
		Stats (T2 and T4)		
		Stats (T2 and T5)		
		Stats (T3 and T4)		
		Stats (T3 and T5)		
		Stats (T4 and T5)		

The procedure shown in Table 2 describes the 14 steps conducted by the GEOTrat - Points tool when the user starts running it. The first step consists of reprojecting the point layers into a projected coordinate system, since ordinary kriging will be performed in subsequent steps. This technique requires the data to be in a metric coordinate system so that the semivariograms, calculated considering the distance between the samples, can be calculated, and reprojecting the input data guarantees suitability for the processes conducted.

Then, in step 2, the relevant fields in the points layer, which represent the agricultural variable of interest and treatment specification, are standardized with a specific name. This makes it easier to identify and manipulate the data during the analysis process. In step 3, the points are separated based on the treatment attribute, resulting in separate point layers for each treatment.

In steps 4, 5, and 6, the samples from each treatment are randomly divided. This division is necessary to generate the surface interpolation model using the kriging method and, subsequently, to estimate the error of this model. This method of dividing samples for modeling is known as holdout, a common technique used in machine learning and statistics to evaluate the effectiveness of a mathematical model, and it was chosen due to its ease of implementation. In this method, the dataset is divided into two mutually exclusive subsets: a training set used to generate the estimation model (usually 80% of the data), and a test set used to evaluate the model’s performance (usually 20% of the data). More information on the holdout technique can be found in [13], and more recently in the works by [14] and [15].

In step 7, interpolation by ordinary kriging is conducted using 80% of the points with samples from each treatment, estimating the variable of interest for the study area. This results in the creation of estimated surfaces of the variable of interest based only on the points of each treatment (T1, T2, T3, T4, T5). Kriging is a technique widely used in geostatistics and spatial analysis to estimate unknown values at unsampled locations based on known point observations [16]. This technique is used in agricultural applications to map the spatial variability of soil attributes, crop yields, and other agricultural parameters [10,17,18].

Ordinary kriging stands out as one of the most widely used techniques in geostatistics due to its ability to consider the spatial dependence of data. This approach assumes that the correlation between values at different locations can be modeled using a semivariogram, which describes how the variability of the data varies with the distance between the sampled points. It then estimates unsampled values by weighting the closest observations and the spatial correlation structure [19].

In the GEOTrat - Points tool, the parameters used to run the ordinary kriging algorithm, provided by the SAGA tester, are the sample points from the treatments and the parameters defined in the input, which are the variable of interest, contour of the study area, size of the interpolation pixel, and semivariogram model. To facilitate comparisons of the experiments, the other parameters were kept at the algorithm’s default values (lag equal to 100, skip equal to 1, global search range, maximum search distance equal to 1,000, minimum of 16 neighbors, and maximum of 20 neighbors).

In steps 8 and 9, the estimated surfaces are sampled using the 20% of the points separated in step 6. After this sampling, the estimation error is calculated by subtracting the estimated value of the variable of interest from the corresponding measured value. This calculated error, in step 10, is used to generate a surface that represents the error calculated for the surfaces. This step is conducted again using the ordinary kriging technique. Then, in step 11, the agricultural variable estimation surface for each treatment and its respective error surface are added together, producing final estimated surfaces for the treatments. In step 12, these final surfaces are cut out, thus limiting the analysis to the space of the study area.

Finally, in step 13, the final surfaces of the treatments are subtracted, allowing a comparison between the treatment defined as the reference and the other treatments. In addition, in step 14, basic statistics are calculated for the surfaces resulting from the subtractions, providing important information on the differences between the treatments, such as sum, mean, standard deviation, maximum, and minimum.

### Outputs

The outputs generated by the resource developed in this study consist of estimated surfaces in matrix format for the study area of the agricultural variable of interest, taking into account the treatments. The T1 surface represents an estimate of the agricultural variable for the study area if it were treated exclusively with the T1 treatment, and the same applies to the T2, T3, T4, and T5 outputs.

In addition, the tool generates an output called *Gain*, which is a surface in matrix format representing the gain associated with the treatment defined as a reference in relation to the other treatments. An output file with an HTML extension, entitled *Statistics of Gain*, is generated to present the basic statistics of these surfaces. It should be noted that the results generated are presented in the same unit of measurement as the agricultural variable of interest, and the execution time is variable, depending on the settings of the equipment used and the number of points to be analyzed.

## Performance evaluation and discussions

This topic presents the performance evaluation of the GEOTrat - Points tool. The data used for the case study were provided by the company Lallemand Plant Care Ltda (in the study, no experiments were carried out with live plants). They belong to experiments that followed a block design, consisting of two different treatments with products of biological origin. It is important to note that information on the specifications of the products used is confidential and therefore not included in the scope of this research.

The simulated surfaces for the experimental area under treatments T1 and T2 were evaluated using the Root Mean Square Error (RMSE), calculated from samples taken from the database prior to the case study. RMSE is a metric widely used to assess the accuracy of forecasting and estimation models. The lower the RMSE value, the smaller the relative discrepancy between the estimates and the values measured in the field. Equation 1 shows the formula used to calculate the RMSE as a percentage:


$$RMSE\;(\%)=\;\frac{\sqrt{\frac{{\sum \left(y_{i}-{\hat{y}}_{i}\right)}^{\text{2}}}{n}}}{\frac{\sum y_{i}}{n}}*\;\text{1}\text{0}\text{0}$$ (1)

where $y_{i}$ represents the yield value measured in the field; ${\hat{y}}_{i}$ represents the estimated yield value; and n the number of evaluation samples.

Another measure used to evaluate the surfaces calculated in this study was Pearson’s Correlation Coefficient (r), which can take values in the range −1 to +1. This coefficient was used to measure the intensity and direction of the linear relationship between the yield values measured in the field and the values estimated for the T1 and T2 surfaces. Equation 2 shows the formula used to calculate r:


$$r=\;\frac{\sum \left(y_{i}-\;\overset{―}{y}\right)\left({\hat{y}}_{i}-\overset{―}{\hat{y}}\right)}{\sqrt{\left(\sum {\left(y_{i}-\;\overset{―}{y}\right)}^{\text{2}}\right)\left(\sum {\left({\hat{y}}_{i}-\overset{―}{\hat{y}}\right)}^{\text{2}}\right)}}$$ (2)

where $y_{i}$ represents the yield measured in the field; $\overset{―}{y}$ represents the average yield measured in the field; ${\hat{y}}_{i}$ represents the estimated yield value; and $\overset{―}{\hat{y}}$ represents the average of the estimated productivity.

While the case study demonstrates the tool’s effectiveness, the data used were provided by a single source. Future work will include data from a wider range of sources and experimental conditions to further validate the tool’s applicability across diverse agricultural contexts. Even though the results are promising, broader validation across diverse crops, environments, and experimental designs is necessary to fully establish the generalizability and reliability of GEOTrat - Points.

### Data

**Fig 2:**
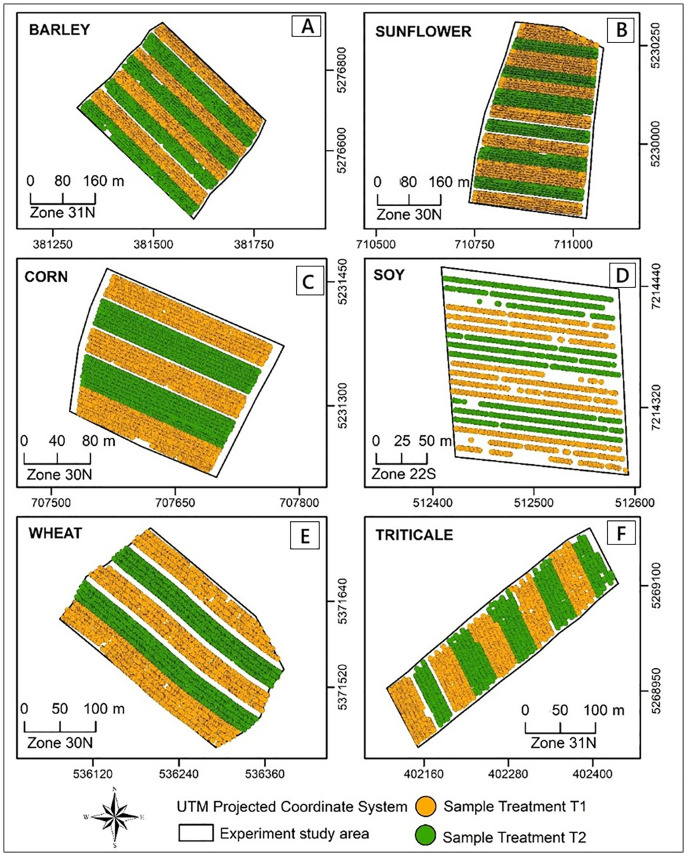
Location map of the experimental areas for the case study using GEOTrat – Points.

The samples were collected from six experimental areas, five of them located in France and one in Brazil. In these areas, different grain crops were grown, including barley, sunflower, corn, soybeans, wheat, and triticale. Fig 2 shows the geographical location of these six areas as well as the configuration adopted for the treatments’ design.

The map in Fig 2. shows the spatial distribution of the samples from treatments T1 and T2. The experiments aimed to assess the impact of the T2 treatment on increasing the yield of the crops under study. To this end, the samples were collected using a combine harvester, to cover the entire experimental area homogeneously. It should be added that the samples are already collected in a georeferenced manner, regardless of the direction of harvest or layout of the treatments. The presence of empty areas in some treatment strips is due to faults in the planting line or errors in the machine’s yield measuring equipment.

Two vector files in shapefile format were made available for each of the areas: a vector file with point-type geometry, with a numeric field containing the yield value in kg/ha and another binary textual field specifying the treatment used, identified as T1 or T2; and another vector file of polygon geometry representing the delimitation of the experimental area. Table 3 shows relevant information about each area, including the crop, size of the area, and basic statistics relating to the total yield samples and for each treatment.

**Table 3:** Crop, area size, and basic statistics of the yield samples.

Cultivation	Area size (ha)	Treatment	Number of samples	Average (kg/ha)	Sum (kg/ha)	Minimum (kg/ha)	Maximum (kg/ha)	Standard Deviation (kg/ha)	Coefficient of variation (%)
Barley	11.48	Total	4,800	5600.00	26869700.00	1000.00	10100.00	1530.00	27.32
		T1	2,400	5680.00	13635900.00	1200.00	10100.00	1570.00	27.64
		T2	2,400	5510.00	13233800.00	1000.00	9900.00	1480.00	26.86
Sunflower	12.62	Total	9,800	3591.52	35196923.10	2132.10	4829.00	494.26	13.76
		T1	4,900	3468.91	16997649.60	2132.10	4827.20	503.90	14.53
		T2	4,900	3714.14	18199273.50	2138.80	4829.00	452.32	12.18
Corn	3.92	Total	4,400	10362.17	45593563.00	5364.20	15072.20	1362.58	13.15
		T1	2,200	10436.65	22960630.90	5364.20	15072.20	1458.12	13.97
		T2	2,200	10287.69	22632932.10	5670.70	15000.20	1255.40	12.20
Soy	3.2	Total	1,600	4470.00	7156520.00	3840.00	5090.00	250.00	5.59
		T1	800	4450.00	3557810.00	3840.00	5070.00	250.00	5.62
		T2	800	4500.00	3598710.00	3880.00	5090.00	240.00	5.33
Wheat	5.01	Total	3,400	8060.00	27420830.00	4780.00	10900.00	980.00	12.16
		T1	1,700	7760.00	13191740.00	4950.00	10610.00	920.00	11.86
		T2	1,700	8370.00	14229080.00	4780.00	10900.00	950.00	11.35
Triticale	3.54	Total	1,940	6163.93	11958025.90	2520.80	9607.00	1181.11	19.16
		T1	970	6063.13	5881237.70	2520.80	9607.00	1215.33	20.04
		T2	970	6264.73	6076788.20	2682.20	9326.70	1136.97	18.15

Table 3 provides an analysis of the yield of the different agricultural crops and in relation to the treatments. The case study included various grain crops with different area sizes, ranging from 3.20 ha to 12.62 ha. In addition, the number of samples differed considerably, with intervals ranging from 1,600 points for the soybean crop to 9,800 points for the sunflower crop, with the number of samples being proportional to the size of the area and considering the planting lines used to conduct the experiment.

The number of samples per hectare recommended for the use of kriging applied to agriculture varies according to spatial variability, area size, desired precision, and available resources [20]. In the experiments in this research, the ratio of the number of samples per area is significantly high, with the barley crop having the lowest number of samples per area, approximately 418.12 samples/ha, and the maize crop having the highest number of samples per area, approximately 1,122.45 samples/ha. The equipment used to collect yields for the experimental areas provides a high density of samples.

The average yield of the experimental areas ranges from 3,591.52 kg/ha for the sunflower crop to 10,362.17 kg/ha for the corn area. The analysis of the sum of yields shows that maize was the crop with the highest yield, at 45593563.00 kg/ha, while soya recorded the lowest yield, at 7156520.00 kg/ha. In terms of the percentage of variation in yield values, barley and triticale have the greatest variability, with coefficients of variation of 27.32% and 19.16%, respectively. The sunflower, corn, and wheat crops show similar variability, with values of 13.76%, 13.15% and 12.16%, respectively, while the soybean crop has the lowest variability, with 5.59%.

Table 3 includes the yield averages for each treatment in each experiment, showing significant differences between the yield averages. The discrepancies between the average yields of the treatments range from 50 kg/ha for the soybean crop to 610.10 kg/ha for the wheat crop. It is important to note that, in all the experiments, the reference treatments were those of treatment T2; however, higher yield averages were noted in treatment T1 for the barley, corn, and soybean crops.

Variability in grain crop yields is a phenomenon that can be linked to various factors. Fluctuations in rainfall, temperature, and weather patterns affect the growth and development of these crops. In addition, the physical and chemical characteristics of the soil, such as texture, fertility, and acidity, directly interfere with the absorption of nutrients by plants. Another important factor is the choice of agricultural management, such as the use of seed varieties, fertilizers, irrigation, and pest and disease control. The interaction between these factors can lead to variations in crop yield [21].

### Parameters

The GEOTrat - Points case study was preceded by a sample partitioning stage, aimed at external evaluation of the yield estimates generated by the models. To do this, 20% of the total samples were randomly selected for each treatment. This process was conducted using the QGIS software, making use of the random selection functionality.

The input parameters for execution were defined as the remaining 80% of the samples after the initial partitioning, selection of the field containing the yield, selection of the field specifying the treatment, and definition of the reference treatment set as T2. In addition, the boundaries of the experimental areas were included, in addition to the projected coordinate system referring to the location of each area, a standard pixel size of 1.50 meters, and the selection of the semivariogram model with linear equation as standard.

## Results

**Fig 3:**
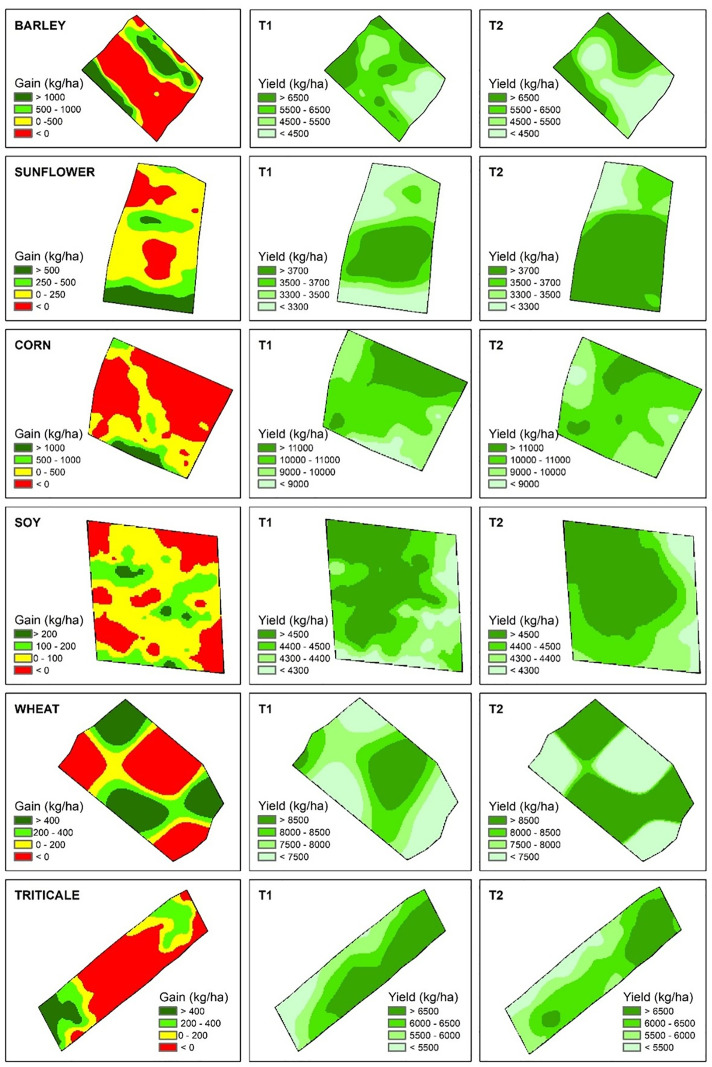
Spatial distribution maps of yield gain and yield for different crops (barley, sunflower, corn, soy, wheat, and triticale) using kriging.

Fig 3 shows spatial distribution maps of yield gain and yield for different crops (barley, sunflower, corn, soy, wheat, and triticale) using kriging techniques. For each crop, three maps are presented: one of yield gain and two of yield under two treatments (T1 and T2).

For barley, the yield gain map (kg/ha) shows significant areas with negative (red) and positive (green) gains, indicating variability in the treatment’s impact. The areas of highest gain are concentrated in certain regions, suggesting that the treatment is effective in a non-uniform manner. The yield maps under T1 and T2 show a more homogeneous distribution compared to the gain. Regions of higher yield (>6500 kg/ha) are consistently green, while areas of lower yield (<4500 kg/ha) are less prevalent. T2 seems to have a slightly better yield distribution than T1.

For sunflower, the yield gain map (kg/ha) shows a predominance of positive gains (green) and small to moderate gains (yellow), with fewer areas of negative gain, suggesting a more uniformly positive effect of the treatment. The yield maps for T1 and T2 show a homogeneous yield distribution. Areas with higher yield (≥3700 kg/ha) are well distributed, with T2 showing slightly better yield.

In the case of corn, the yield gain map (kg/ha) reveals significant variability, with areas of negative gain (red) and significant positive gain (green). Areas with high gain (>1000 kg/ha) are well distributed, but losses are also significant. The yield maps under T1 and T2 show high yield quantities (>11000 kg/ha) in various regions, with T2 having a somewhat more uniform distribution of high yields compared to T1.

For soy, the yield gain map (kg/ha) shows mainly positive gains (green and yellow), with few areas of negative gain, and the gain distribution is relatively uniform. The yield maps for T1 and T2 show yield above 4500 kg/ha generally, with T2 showing a slightly improved distribution compared to T1.

Regarding wheat, the yield gain map (kg/ha) indicates considerable variability with large areas of negative gain (red) and positive gain (green). Positive gain areas are less dominant, suggesting that the treatment may not be uniformly effective. The yield maps for T1 and T2 show regions with yield ranging from <7500 kg/ha to >8500 kg/ha, with T2 showing a slightly more uniform and higher yield distribution compared to T1.

For triticale, the yield gain map (kg/ha) shows alternation between areas of high gain (>400 kg/ha) and loss (<0 kg/ha), with positive gain areas being more dominant but with considerable variability. The yield maps for T1 and T2 show yield ranging from <5500 kg/ha to >6500 kg/ha, with T2 showing a better spatial distribution of high yields compared to T1.

Visually, the yield gain maps for barley and corn showed yield losses, which align with the basic statistics of the samples presented in Table 3, as the yield mean of these crops was lower in the reference treatment. This also justifies the outcomes for sunflower and soy crops, where there is yield gain across almost the entire experimental area.

Table 4 shows the results of the basic statistics of the yield gain surface, giving the user a better understanding of the estimate made.

**Table 4:** Basic statistics of the yield gain surface.

Cultivation	kg/ha				
	Minimum	Maximum	Sum	Average	Standard deviation
Barley	−2818.89	3071.81	−3961719.13	−138.01	1185.83
Sunflower	−318.47	918.32	6669211.02	211.34	248.49
Corn	−1534.31	2251.93	−1382874.80	−141.14	576.46
Soy	−185.59	248.82	301334.48	37.63	82.74
Wheat	−12134.21	9171.82	6356454.12	507.38	5122.48
Triticale	−913.61	809.09	−594242.22	−67.06	374.54

Table 4 shows the maximum, minimum, sum, average, and standard deviation of the yield gain surface. Based on these metrics, it is possible to conduct a general analysis of yield loss or gain and conclude whether the treatment was efficient. The results indicate that the wheat crop had a wider range of yield values, varying from a loss of −12134.24 kg/ha to a gain of 9171.82 kg/ha. In terms of sum productivity, barley showed the greatest loss, with a negative balance of 3961719.13 kg/ha, whereas sunflower showed a yield gain of 6669211.02 kg/ha.

In this study, each crop had a different number of samples for evaluation. Table 5 shows the RMSE (%) and Pearson’s correlation coefficient for each treatment surface.

**Table 5:** RMSE and r of the estimates for T1 and T2.

Cultivation	Number of samples	RMSE (%)		r	
		T1	T2	T1	T2
Barley	480	14.13	23.27	0.86	0.68
Sunflower	980	9.93	8.12	0.75	0.76
Corn	440	9.78	9.98	0.72	0.62
Soy	160	4.92	4.6	0.68	0.58
Wheat	340	13.84	69.71	0.21	−0.16
Triticale	194	17.13	16.45	0.52	0.52

Table 5 shows the RMSE in percentage of the estimates generated for the T1 and T2 surfaces. The analysis of yield surface simulation accuracy results for different crops, considering two types of treatment (T1 and T2) and utilizing kriging technique, reveals significant insights into prediction precision and treatment effectiveness.

For barley, treatment T1 exhibited an RMSE (Root Mean Square Error) of 14.13% and a correlation coefficient (r) of 0.86, while T2 had an RMSE of 23.27% and an r of 0.68. This suggests that treatment T1 had a more precise simulation and a stronger correlation between simulated and observed values compared to T2. In the case of sunflower, both treatments showed relatively low RMSE, with 9.93% for T1 and 8.12% for T2. Additionally, both treatments demonstrated similar correlation with observed values, with r of 0.75 for T1 and 0.76 for T2. This indicates that both treatments were effective in simulating sunflower productivity, with good precision and correlation.

For corn, treatment T1 had an RMSE of 9.78% and an r of 0.72, while T2 had an RMSE of 9.98% and an r of 0.62. Although both treatments showed similar precision, T1 exhibited a slightly stronger correlation with observed values compared to T2. Soybean showed consistent results between treatments T1 and T2, with RMSE of 4.92% and 4.6%, respectively, and a moderate correlation between simulated and observed values (r = 0.68 for T1 and r = 0.58 for T2).

For wheat, treatment T1 had an RMSE of 13.84% and an r of 0.21, while T2 exhibited a much higher RMSE of 69.71% and a negative correlation (r = −0.16). This indicates that wheat simulation was less precise, especially for treatment T2, where there was an inverse correlation between simulated and observed values. Both treatments T1 and T2 for triticale showed similar RMSE, with 17.13% for T1 and 16.45% for T2. Additionally, both treatments demonstrated identical correlation with observed values, with r of 0.52 for both. This suggests that both treatments were equally effective in simulating triticale productivity.

## Discussions

Overall, the application of treatments shows variable effects depending on the crop. Some treatments, particularly T2, tend to present a more uniform and, in some cases, superior yield distribution. The variability in yield gain indicates that the treatment’s effectiveness can be highly dependent on local conditions. Visual analysis of these spatial distributions can be useful for directing more precise and efficient agricultural practices.

The variability in spatial distribution patterns of yield gain across crops can be attributed to a complex interplay of biotic and abiotic factors, as well as the spatial interpolation techniques used. In barley, variations in soil, water availability, and local climatic conditions can influence the crop’s response to the applied treatment [22]. Additionally, biotic factors such as the presence of specific pests or diseases in certain regions may interact complexly with treatments, leading to a non-uniform crop response [23].

For sunflower, the more uniform distribution of yield gain can be attributed to the crop’s ability to adapt to a variety of soil and climate conditions [24]. The kriging technique, by effectively capturing the spatial variability of data, can produce smoother and more homogeneous gain surfaces [25].

Regarding corn, significant variability in spatial distribution patterns of yield gain may be explained by a combination of factors, including soil variations, topography, nutrient availability, and biotic stresses [26]. Studies suggest that kriging can help identify complex spatial patterns in the data, allowing for a better understanding of the interactions among these factors [27].

In the context of soybeans, the relatively uniform distribution of yield gain may be explained by the crop’s ability to adapt to a wide range of environmental conditions [28]. Kriging can be effective in capturing spatial patterns in soybean productivity, allowing for a more precise analysis of the effects of applied treatments [29].

For wheat, the considerable variability in spatial distribution patterns of yield gain suggests a complex interaction between biotic and abiotic factors [30]. Studies indicate that kriging can be useful in identifying spatial patterns in wheat yield and understanding the underlying causes of variability [31]. These analyses underscore the importance of considering a variety of biotic, abiotic factors, and spatial interpolation techniques in interpreting spatial distribution patterns of yield gain in different crops.

The relationship between the performance of kriging algorithms and crop type is a crucial aspect to consider when analyzing the accuracy of yield simulations. The spatial distribution of each crop and its interaction with the environment can significantly influence the precision of the algorithms. Recent studies suggest that different crops have distinct spatial patterns and respond differently to biotic and abiotic conditions, which can affect the effectiveness of spatial interpolation techniques like kriging.

For barley, the presence of soil variations, water availability, and local climatic conditions can influence the crop’s response to the applied treatment [22]. The interaction between these factors and the spatial distribution of barley can create complex variability patterns that kriging needs to capture to provide accurate predictions. The lower accuracy observed in treatment T2 for barley may be attributed to greater spatial heterogeneity not adequately captured by the model.

In the case of sunflower, its ability to adapt to various soil and climatic conditions can result in a more homogeneous spatial distribution of yield gains [24]. This homogeneity makes it easier for kriging to interpolate the data with higher precision, resulting in lower RMSEs and stronger correlations, as observed in treatments T1 and T2.

For corn, the significant variability in spatial distribution patterns of yield gains can be explained by a combination of factors, including soil variations, topography, nutrient availability, and biotic stresses [26]. These variables create a complex scenario for kriging, resulting in varying precision between treatments. Kriging’s ability to capture this complexity directly influences the accuracy of the predictions.

Soybeans, on the other hand, show a relatively uniform distribution of yield gains, attributed to their ability to adapt to a wide range of environmental conditions [28]. This spatial uniformity facilitates data interpolation, resulting in low RMSEs and moderate correlations, demonstrating kriging’s effectiveness under conditions of lower variability.

For wheat, the considerable variability in spatial distribution patterns of yield gains suggests a complex interaction between biotic and abiotic factors [30]. The low correlation and high RMSE observed in treatment T2 may indicate kriging’s inadequacy in capturing highly variable spatial patterns influenced by multiple environmental factors.

Finally, for triticale and other analyzed crops, the effectiveness of kriging depends on the algorithm’s ability to capture spatial heterogeneity and complex interactions between the crop and the environment. Studies indicate that advanced kriging techniques, such as co-kriging and kriging with auxiliary variables, can improve prediction accuracy in high variability scenarios [27,31].

### Implications of the tool: Comparison, applicability, and challenges

The GEOTrat - Points tool stands out from conventional approaches in agricultural experiment analysis by integrating geostatistical techniques and spatial modeling to evaluate treatment differences. Traditional methods, such as analysis of variance (ANOVA) and mean comparison tests, assume spatial independence among experimental units and often overlook the inherent spatial variability in field conditions. This limitation can lead to inaccurate conclusions, particularly in experiments where treatment effects are subtle and influenced by heterogeneous environmental conditions. In contrast, the geostatistical interpolation used in GEOTrat - Points enables a continuous spatial representation of the variable of interest, highlighting patterns that might not be evident in traditional statistical approaches. This advantage is particularly relevant to precision agriculture, where decision-making relies on a detailed understanding of the spatial distribution of treatment effects.

The tool is most appropriate for agricultural experiments where georeferenced data is collected at high sample densities, allowing for effective geostatistical interpolation. Large-scale experiments, such as randomized block or strip trials, can significantly benefit from spatial mapping of treatment responses. However, for experiments with a limited number of samples or irregular distribution, the accuracy of the estimates may be compromised. Additionally, the tool is especially useful for crops with high spatial variability in yield, such as corn and wheat, where subtle treatment differences can be difficult to detect using conventional statistical analyses.

Despite its advantages, GEOTrat - Points has certain limitations that should be considered. The accuracy of estimates depends on the quality and quantity of input data, requiring a minimum number of sampling points for reliable geostatistical interpolation. Furthermore, the choice of semivariogram model directly affects the quality of the estimated surfaces, which may require technical expertise for proper selection. Another limitation is that the tool currently operates only with ordinary kriging interpolation, which may not be suitable for all agricultural variables. Alternative methods, such as universal kriging or machine learning-based models, could be explored in future versions to expand the tool’s applicability.

The adoption of tools like GEOTrat - Points can significantly contribute to the modernization of agricultural experiment analysis, enabling a more detailed and visually intuitive approach. However, it is essential for researchers and practitioners to understand the ideal conditions for applying the tool, ensuring that the generated analyses are correctly interpreted. Future improvements may include incorporating cross-validation techniques to enhance the calibration of geostatistical models, expanding support for different interpolation methods, and integrating remote sensing data. These advancements could make GEOTrat - Points an even more robust solution for spatial mapping and analysis in agricultural experimentation.

### Limitations and future developments

The effectiveness of kriging in simulating yield gain surfaces varies significantly across different crops, influenced by their spatial distribution and interaction with environmental factors. While automation offers substantial benefits in terms of efficiency and consistency, it also presents challenges in handling complex variability. Advanced kriging techniques and careful consideration of crop-specific conditions are essential for improving prediction accuracy in automated systems.

One significant advantage of automating yield gain surface simulation through a QGIS plugin is efficiency and speed. Automated processes allow for the rapid generation of spatial distribution maps, significantly reducing the time and resources required compared to manual methods. This is particularly beneficial in large-scale agricultural studies where timely decision-making is crucial.

Another advantage is consistency. Automated processes minimize human error, ensuring that kriging is applied uniformly across different datasets and crops. This consistency enhances the reliability of the results, as the same methodology is systematically used, reducing the potential for variability introduced by different operators. Additionally, automation increases accessibility. Automated tools make advanced geostatistical methods, such as kriging, available to users who may not have extensive expertise in spatial analysis. This democratization of technology enables a broader range of stakeholders, including farmers and agronomists, to utilize sophisticated techniques for yield optimization.

Despite these benefits, there are notable disadvantages to consider. One major drawback is the difficulty in handling complexity. Automated kriging may struggle with highly variable and complex spatial patterns, as observed in crops like barley and wheat. These cases may require manual adjustments or the use of advanced kriging techniques to achieve better accuracy, which can be challenging to implement in an automated system.

The accuracy of automated kriging is also highly dependent on data quality. Sparse or poor-quality input data can lead to inaccurate predictions, undermining the reliability of the generated yield gain surfaces. Ensuring high-quality and sufficiently dense data is critical for the success of automated spatial analysis.

Finally, customization limitations can be a significant disadvantage. Automated processes might lack the flexibility to tailor kriging parameters to specific crop conditions or unique spatial patterns. This limitation can hinder the ability to achieve the most accurate and relevant results for agricultural contexts.

GEOTrat - Points is a resource for QGIS that allows to evaluate the efficiency of agricultural treatments from field experiments. One of the tool’s limitations is the need for the user to define the semivariogram model to be used for ordinary kriging. This definition has an impact on the accuracy of the models generated. Another limitation is that only the ordinary kriging method is available, and although it allows for the generation of high-quality maps, it does not necessarily work well in every conceivable application or for any variable. Ideally, more robust machine learning algorithms such as Support Vector Machine (SVM), Artificial Neural Networks (ANN), and Random Forests (RF) should be used.

Future developments include automatic optimization of the semivariogram model to adjust the ordinary kriging models, the inclusion of cross-validation techniques to evaluate the model, and finally, an extension of the GEOTrat resource that will make it possible to use image bands or vegetation indices obtained from different sources to evaluate field treatments. Other future developments will include the integration of advanced interpolation methods, such as co-kriging and machine learning algorithms (e.g., Support Vector Machines, Artificial Neural Networks, and Random Forests), to handle more complex spatial patterns and improve prediction accuracy. Also, to reduce user dependency and improve consistency, future versions of GEOTrat - Points will include automated optimization of the semivariogram model and other critical parameters, ensuring more robust and reliable results.

## Conclusions

To enable a quantitative and spatial analysis of agricultural management, the GEOTrat - Points tool was developed and integrated with the QGIS software. This resource models variables collected in the field, performs statistical analyses, and generates map visualizations. The approach used is based on the ordinary kriging method, allowing the variables of interest to be simulated across the entire experimental area based on individual treatments. Subsequently, a comparison is made between the reference treatment and the other treatments. The results are exported in raster format, and the statistics are available in the form of an HTML file.

To evaluate the performance of GEOTrat - Points, a case study was conducted which involved comparing experimental areas where two different treatments were applied to crops such as barley, sunflower, corn, wheat, soybeans, and triticale. The study considered different sample sizes of agricultural yield in the areas under analysis. In general, the simulations under the different treatments showed high levels of mapping accuracy, providing a qualitative and quantitative interpretation of yield variations.

The accuracy of kriging algorithms in simulating agricultural yield is significantly influenced by the crop type and its spatial distribution. The ability of crops to interact with the environment and the spatial heterogeneity of environmental factors play a crucial role in the effectiveness of spatial interpolation techniques. To improve prediction accuracy, it is essential to consider these variables and possibly integrate advanced kriging techniques that can better handle the spatial complexity of agricultural data.

GEOTrat - Points offers the flexibility to evaluate agricultural experiments of varying sizes. The accuracy of the estimates generated is directly related to the quality, quantity, and nature of the variables collected in the field. The data interpretation capacity provided by this tool significantly contributes to agricultural experimentation, helping in the selection of appropriate management practices and in understanding the most effective treatments.

GEOTrat - Points represents a significant advancement in the evaluation of agricultural experiments, offering a free, open-source solution for spatial and statistical analysis. Its integration with QGIS makes it accessible to a wide range of users, from researchers to farmers, and has the potential to improve decision-making in agricultural management.

**PREPRINT:** A preprint has been published previously Xavier et al., 2024 [32].
